# Methylenetetrahydrofolate reductase (MTHFR) polymorphisms and promoter methylation in cervical oncogenic lesions and cancer

**DOI:** 10.1111/jcmm.12032

**Published:** 2013-02-28

**Authors:** Anca Botezatu, Demetra Socolov, Iulia V Iancu, Irina Huica, Adriana Plesa, Carmen Ungureanu, Gabriela Anton

**Affiliations:** a Viral Genetic Engineering Laboratory, Romanian Academy “Stefan S. Nicolau” Virology InstituteBucharest, Romania; b Obstetrics and Gynecology Department, “Grigore T. Popa” University of Medicine and PharmacyIassy, Romania; c Pathology Department, “Grigore T. Popa” University of Medicine and PharmacyIassy, Romania

**Keywords:** MTHFR polymorphism, hrHPV, cervical cancer, DNA methylation

## Abstract

The aim of this study was to investigate the role of methylenetetrahydrofolate reductase (MTHFR) polymorphisms and MTHFR methylation pattern in cervical lesions development among women from Romania, a country with high prevalence of human papillomavirus (HPV) cervical infections. To achieve this goal, blood samples and cervical cytology specimens (*n* = 77)/tumour tissue specimens (*n* = 23) were investigated. As control, blood and negative cytological smears (*n* = 50) were used. A statistically significant association was found between *T* allele of *C677T* polymorphism and cervical lesions, heterozygote women presenting a threefold increased risk (normal/cervical lesions and tumours: wild homozygote 34/41 (0.68/0.41), heterozygote 14/51 (0.28/0.51), mutant homozygote 2/8 (0.04/0.08); OR = 3.081, *P* = 0.0035). Using χ square test for the control group, the HPV-negative and HPV-positive patients with cervix lesions, a significant correlation between viral infection and *T* allele of *C677T* polymorphism (*P* = 0.0287) was found. The MTHFR promoter was methylated in all HGSIL and tumour samples, significant differences being noted between HPV-positive samples, control group and cases of cervical dysplastic lesions without HPV DNA (*P* < 0. 0001) and between samples from patients with high-risk (hr)HPV versus low-risk (lr)HPV (*P* = 0.0026). No correlations between polymorphisms and methylation were observed. In Romania, individuals carrying *T* allele are susceptible for cervical lesions. MTHFR promoter methylation is associated with cervical severity lesions and with hrHPV.

## Introduction

Although cervical cancer is a model for early cancer detection, it remains one of the most important causes of mortality affecting women worldwide. Molecular and epidemiological data proved that human papillomavirus (HPV) is the aetiological agent of cervical cancer 1. Many women are infected with HPV, but only a minority develop cervical cancer; therefore other factors like HPV genotypes, multiple sexual partners, early start of sexual life, multiple pregnancies, diet, smoking and oral contraceptives 2–5 are linked with cervical oncogenesis. Among host factors, immunomodulatory and metabolic pathways might be determinants for the susceptibility to the development of cervical neoplasia or cancer.

In this context, some investigations have been focused on the role of folate in carcinogenesis, including cervical one 6. Folate is essential for the synthesis of nucleotides, its deficit inducing double-strand breaks and increased cancer risk. Low levels of folate correlate with alterations in cell replication, DNA excision and repair and DNA methylation pattern 7. The metabolism of folate is regulated by methylenetetrahydrofolate reductase (MTHFR), an enzyme that catalyses the reduction of 5,10-methylenetetrahydrofolate (5,10-methyleneTHF) to 5-methylTHF, the methyl donor for methionine synthesis from homocysteine 8. DNA methylation also depends on methyl-group donor S-adenosylmethionine (SAM). The methyl group used by SAM for methylation reactions is mainly derived from folate metabolism 9.

Although multiple polymorphisms in MTHFR gene were described, epidemiologic studies have shown that only two of them affect the enzymatic activity and are related to different disorders including malignancies 10–12. These polymorphisms are *C677T* (which results in an alanine-to-valine substitution) and *A1298C* (leading to the replacement of glutamine by alanine). *A1298C* polymorphism has a lower effect in reducing enzyme activity, compared with the C*677T* mutation, which determined different enzyme thermolability degree (assessed as residual activity after heat inactivation). The enzyme residual activity is almost 18–22% in *677TT* individuals compared with heterozygous *677CT* (56%) and normal *677CC* (66–67%) 13, 14.

Whereas *677CT* heterozygote patients were reported to have mild elevation levels of plasma homocysteine and non-methylated folate in red blood cells (both conditions involving impairment in 5-methylTHF synthesis) 14, *677TT* genotype was frequently associated with different types of cancer (colon cancer, hepatocellular carcinoma) and cardiovascular diseases 14–16. On the other hand, *A1298C* polymorphism correlates with ovarian cancer and lymphoblastic acute leukaemia 17–19.

It is already known that MTHFR polymorphisms can modulate DNA methylation status and the alteration of DNA methylation pattern is associated with carcinogenesis 20. Maintaining the DNA methylation patterns is a major feature of epigenetic control on genome stability and gene transcription regulation 21. In addition, the MTHFR gene may be subjected to promoter CpG island methylation. To our knowledge, little is known regarding the involvement of MTHFR gene promoter methylation in cancer. The MTHFR promoter was found to be hypermethylated in renal tumours 22 and in male infertility 23. Sun *et al*. performed an integrated analysis of gene expression related to CpG islands methylation. They concluded that differentially expressed genes which exhibited differential methylation pattern of one or more CpG islands within the region promoter of the gene, presented an inversely correlation between mRNA abundance and CpG methylation status 24. On the other hand, reduced expression of metabolic enzyme genes, including MTHFR, was significantly associated with oesophageal squamous cell carcinoma 25.

This study aimed to investigate MTHFR polymorphisms and the methylation pattern of MTHFR promoter in relationship with susceptibility to cervical lesions and cervix papillomaviruses infection in Romania, which has one of the highest rates of cervical cancer in Europe (incidence 33.9/100,000; mortality 16.9/100,000 – Romanian Statistic Annual 2004).

## Materials and methods

### Sample collection

Investigated patients were selected based on suggestive cervical pathology for human papillomavirus infection. Cervical cytology specimens (*n* = 77) from women with dysplastic cervical lesions were collected by a gynaecologist, and conventional cytological screening or histological exams of the samples were performed by a trained pathologist. According to cytological investigation, the cervical samples were divided as: ASCUS (*atypical squamous cells of undetermined significance*) (27/77, age range: 18–58 years, median: 30), LGSIL (*low-grade squamous intraepithelial lesion*) (27/77, age range: 18–63 years, median: 35) and HGSIL (*high-grade squamous intraepithelial lesion*) (26/77, age range: 25–60 years, median: 36). Tissue specimens (*n* = 23) were obtained from patients with squamous cervical carcinoma (SCC) who underwent surgery for cervix tumour mass removal (age range: 22–69 years, median: 36). Tumours were immediately stored at −80°C.

Control group consisted of women (*n* = 50) with negative cytological smears attending gynaecological clinic for regular investigations (age range: 20–53 years, median: 35). For nucleic acids preservation all samples were collected in sterile tubes with ESwab Collection and Transport System medium (Copan, Brescia, Italy). Blood samples used for the detection of MTHFR polymorphisms were collected on EDTA from all the women enrolled in this study. No age or ethnicity exclusion criteria were used.

### DNA isolation

DNA isolation from blood and cervical samples (smears and tumours) was performed with High Pure PCR Template Preparation Kit (Roche Molecular Biochemicals, Mannheim, Germany), according to manufacturer's recommendations. Isolated DNAs were subsequently stored at −20°. The concentration and purity of each DNA sample was evaluated with NanoDrop spectrophotometer (NanoDropTechnologies, Montchanin, DE, USA).

### HPV genotyping

Human papillomavirus genotyping was performed with Linear Array HPV Genotyping Test (Roche Molecular Biochemicals, Mannheim, Germany), according to the manufacturer's instructions. A pool of biotinylated primers amplify near 450 base pairs from L1 gene of 37 HPV genotypes including 13 high-risk types (16, 18, 31, 33, 35, 39, 45, 51, 52, 56, 58, 59 and 68). An additional primer pair targets the human β-globin gene to provide a control for cell adequacy, extraction and amplification.

### Bisulphite treatment

Bisulphite treatment for unmethylated C residues conversion was realized with EpiTect Bisulfite kit (Qiagen, Valencia, California, USA). Seven-hundred nanogram of each isolated DNA (from cervical swabs and tumours) in a maximum volume of 20 μl was bisulphite treated along with positive (CpGenome Universal Methylated DNA) and negative (CpGenome Universal Unmethylated DNA) controls (Millipore, Billerica, MA, USA).

### Primers

Primers to distinguish methylated and unmethylated status of MTHFR following bisulphite treatments were designed using Methprimer (Li and Dahiya, 2002) and were synthesized by Invitrogen Corporation (Carlsbad, CA, USA):

MTHFR methylated FW 5′-TAGATTTAGGTACGTGAAGTAGGGTAGAC-3′.

MTHFR methylated R 5′-GAAAAACTAATAAAAAACCGACGAA-3′.

MTHFR unmethylated FW 5′-TTTAGGTATGTGAAGTAGGGTAGATGT-3′.

MTHFR unmethylated R 5′-CAAAAAACTAA TAAAAAACCAACAAA-3′.

### Methylation-Specific PCR (MS-PCR)

Methylation-specific PCR was performed in a final volume of 25 μl using Platinum *Taq* DNA Polymerase (1 U), 1× enzyme buffer, 1.5 mM MgCl_2_, 200 μM of each dNTP, 0.3 μM of each specific primers, 5 μl target DNA. The PCR conditions were as follows: 95°C – 5 min., 35 cycles of 95°C – 30 sec., 56°C – 30 sec., 72°C -30 sec. and a final elongation of 72°C – 7 min. The methylated/unmethylated status of the target gene was estimated in 2% agarose gel electrophoresis.

### Direct Q-MSP of Genomic DNA

Direct Q-MSP of genomic DNA was used for evaluating the degree of sample methylation. Standard curves were designed using serial dilution (10 pg, 100 pg, 1 ng and 10 ng) of positive (DNA fully methylated) and negative controls (DNA fully unmethylated), according to Applied Biosystem Guide (Applied Biosystems 7300/7500/7500 Fast Real-Time PCR System, Foster City, CA, USA). The quantity of unmethylated and methylated DNA for each patient sample was extrapolated using the standard curves.

Direct Q-MSP was performed in 50 μl final volume comprising 50 ng/μl DNA, 25 μl FastStart Universal SYBR Green Master (ROX; Roche Molecular Biochemicals, Mannheim, Germany) and 0.3 μM primers. PCR parameters we used were as follows: 95°C/10 min. (1 cycle), 40 cycles at 95°C/15 sec. and 60 sec./specific annealing temperature.

qPCR experiments were performed in duplicate and mean values were used for calculations. To control the quality of DNA samples we used ACTB gene as reference 26.

### Methylation percentage

Methylation percentage was calculated according to method described by Fackler *et al*. (% M = 100× [ng methylated gene A/(ng methylated gene A+ unmethylated gene A)]) 26.

### MTHFR polymorphisms (C677T and A1298C)

Methylenetetrahydrofolate reductase polymorphisms (*C677T* and *A1298C*) were performed in PCR – RFLP technique, according to Frosst *et al*. 14. The primers' sequences for *C677T* mutation were as follows: FW 5′-TGAAGGAGAAGGTGTCTGCGGGA-3′ and R 5′-AGGACGGT GCGGTGAGAGTG-3′ whereas for *A1298C* polymorphism we used: FW 5′-GGGA-GGAGCTGACCAGTGCAG-3′ and R 5′-GGGGTCAGGCCA GGGGCAG -3′. The PCR conditions were as follows: for *C677T* polymorphism, 30 cycles of 94°C/45 sec., 65°C/45 sec., 72°C/45 sec.; for *A1298C* polymorphism, 35 cycles of 94°C/45 sec., 65°C/45 sec., 72°C/45 sec. 198 bp amplicons for *C677T* polymorphism and 138 bp amplicons for *A1298C* polymorphism were subjected to enzymatic restriction with *Hinf I* and *Fnu4HI*, respectively, according to manufacturer's instructions (New England Biolabs). Following digestion, the samples were evaluated in 3% agarose gel electrophoresis. Heterozygote (CT) presented three lanes of 198 pb, 175 bp and 23 bp whereas heterozygote (AC) displayed 138, 119 and 19 bp lanes.

### Statistical analysis

Statistical analysis was performed with GraphPadInstat3. The association between MTHFR polymorphisms and Pap test was analysed using Fisher's exact test, wherever appropriate. The confidence interval was established at 95%. anova (Kruskal–Wallis and Mann–Whitney tests) was used to compare the variance between the investigated groups. The biological samples were provided with patient's agreement and the study protocol was approved by the Ethics Committee.

## Results

### HPV testing

Viral test results for each category of investigated patients are presented in Table 1. In the study group 75/100 patients were HPV positive. The overall prevalence (single and co-infections) of hrHPV genotypes was as follows: 32% HPV16 (16.7% in ASCUS, 7.4% in LGSIL, 42.3% in HGSIL and 65.2% in SCC), 11.8% HPV 31; 9.2% HPV 18; 9.2% HPV 66; 3.9% HPV 33 and 3.9% HPV 51.

**Table 1:** Distribution of overall human papillomavirus (HPV) tested patients

	HPV+ (%)	HPV single infection/total HPV-positive	HPV co-infections/total HPV-positive
ASCUS	15/24 (62.5)	8/15	7/15
LGSIL	19/26 (73)	11/19	8/19
HGSIL	21/27 (77.8)	15/21	6/21
SCC	20/23 (87)	13/20	7/20

### MTHFR polymorphisms and their impact on cervical tumourigenesis

The analysis of MTHFR *C677T* polymorphism in all patients included into the study revealed that 75 cases were homozygote for normal allele (CC), 65 were heterozygote (CT) and 10 were mutant homozygote (TT). The calculated frequency of *C* allele in population was 0.72, whereas the frequency of *T* allele was 0.28. The χ^2^ test was performed with 1 degree of freedom and 5% level of significance (3.84). The value of χ^2^ test was 0.674 (below 3.8), the population being in Hardy–Weinberg equilibrium. The calculated frequency of *A* allele for 1298 polymorphism in our patients was 0.75, whereas the frequency of *C* allele was 0.25. Eighty-one of the investigated patients were normal homozygous (AA), 63 were heterozygous (AC) and 6 were mutant homozygous (CC). The population was in Hardy–Weinberg equilibrium, the value of χ^2^ test being 2.16.

When comparing patients with cervical lesions (including cervical cancer) with the normal group, a statistically significant association was found between *T* allele of *C677T* polymorphism and cervical lesions, heterozygote women presenting a threefold increased risk (normal/cervical lesions and tumours: wild homozygote 34/41(0.68/0.41), heterozygote 14/51(0.28/0.51), mutant homozygote 2/8 (0.04/0.08); OR = 3.081, *P* = 0.0035). By contrast, the MTHFR polymorphism *A1298C* was not associated with cervical disease development (normal/cervical lesions and tumours: wild homozygote 30/51 (0.60/0.51), heterozygote 19/44 (0.38/0.44), mutant homozygote 1/5 (0.02/0.05); OR = 1.441, *P* = 0.2278).

Furthermore, we evaluated a possible link between MTHFR polymorphisms and HPV infection. Using chi-square test for control group, HPV-negative and HPV-positive patients with cervix lesions, we found a significant correlation between viral infection and *T* allele of *C677T* polymorphism (*P* = 0.0287). The results of MTHFR polymorphisms in the investigated groups are presented in Table 2: using chi-square test to compare the MTHFR polymorphisms and HPV type (hrHPV and lrHPV) we found no correlation (*C677T*: wild homozygote 21/5, heterozygote 36/5, mutant homozygote 3/3, *P* = 0.07; *A1298C*: wild homozygote 34/6, heterozygote 22/3, mutant homozygote 3/0, *P* = 0.44).

**Table 2:** Contingency table of the methylenetetrahydrofolate reductase (MTHFR) polymorphisms in control group and in human papillomavirus (HPV)-positive and -negative patients

	MTHFR *C677T*					MTHFR *A1298C*				
						
	CC	CT	TT	χ^2^	*P* value	AA	AC	CC	χ^2^	*P* value
Control group	17	7	1	10.82	0.0287	25	10	0	7.18	0.1267
HPV− cervical lesions	15	9	1			10	13	2		
HPV+ cervical lesions	26	42	7			42	30	3		

### Quantification of MTHFR gene promoter methylation in cervical samples

The methylation percentage (% M) value for negative standard was approximately 0 and no amplification of methylated standard with unmethylated primers was obtained. We validated our data regarding quantitative methylation analysis based on the similar differences between ΔCt values (ΔCt = CtM − CtU) for all dilutions (ΔCt ≍ 0.726). On the other hand the slope value for all the standard curves were around the −3.16 value that means a twofold increase in PCR product per cycle during the linear phase of real-time PCR. The average of calculated efficiencies was around 107.3. The standard curves showed a correlation coefficient (*R*^2^) around 0.999 providing evidence of linearity over the entire range of template concentration.

To eliminate false-positive % M values in cervical samples due to blood cell infiltration/contamination, a cut-off value of 1.569 was established. The cut-off was calculated as the 95th percentile from blood % M values of MTHFR promoter in the blood samples of the patients. We have to mention that some samples presented % M levels below the cut-off.

Using Kruskal–Wallis for MTHFR promoter methylation we noted highly significant differences in median data between different cytological groups (Table 3).

**Table 3:** Methylenetetrahydrofolate reductase (MTHFR) methylation values in the investigated groups

	Range	Median	Mean ± SE	Lower 95% confidence limit	Upper 95% confidence limit	*P* value
Normal	0–10	0	0.8716 ± 0.4685	−0.095	1.839	
ASCUS	0–100	36.07	38.62 ± 6.666	24.83	52.41	<0.0001
LGSIL	0–65.38	15.11	22.08 ± 3.966	13.91	30.25	0.0002
HGSIL	22.98–100	56.15	57.13 ± 4.700	47.47	66.79	<0.0001
SSC	34.55–100	75.01	73.75 ± 4.034	65.39	82.12	<0.0001

Considering positive for methylation all the cervical samples over cut-off, the methylated cases were as follows: 2/25 of control group, 18/24 ASCUS patients and 18/26 LGSIL patients. All HGSIL and tumour samples presented MTHFR promoter methylation, 14/27 HSIL samples and 18/23 tumours displaying more than >60% methylation percentage.

Significant differences in methylation were found between HPV-positive samples, control group and cases of cervical dysplastic lesions without HPV DNA (*P* < 0. 0001). Moreover, statistic differences (*P* = 0.0026) between samples from patients with hrHPV infection versus lrHPV were obtained (Table 4).

**Table 4:** Percentage values of methylenetetrahydrofolate reductase (MTHFR) methylation stratified by human papillomavirus (HPV) presence and oncogenic potential

	Control group	HPV+ cervical lesions	HPV− cervical lesions	hrHPV	lrHPV
Minimum	0	0	0	0	0
Median	0	55.84	39.3	64.54	20.91
Maximum	10	100	100	100	75.79
Mean	0.87	52.38	35.25	57.4	26.82
Std. Error	0.47	3.66	5.14	3.84	7.41
Lower 95% CI of mean	−0.09	45.08	24.66	49.73	10.52
Upper 95% CI of mean	1.82	59.67	45.83	65.08	43.11
Kruskal–Wallis test				Mann–Whitney test	
*P* value	*P* < 0.0001				0.0026

Although, no significant association between MTHFR methylation and MTHFR polymorphisms was noted, we have to mention that CC genotype of *A1298C* polymorphism presented the highest median value (Table 5).

**Table 5:** Calculated methylenetetrahydrofolate reductase (MTHFR) methylation values for the investigated MTHFR polymorphisms

Polymorphism	*C677T*			*A1298C*		
		
Genotype	CC	CT	TT	AA	AC	CC
Minimum	0.0	0.0	0.0	0.0	0.0	35.38
Median	42.34	47.79	50.09	42.16	47.37	69.62
Maximum	100.0	100.0	100.0	100.0	100.0	99.85
Mean	43.16	50.60	48.74	46.30	46.64	65.22
SE	4.63	4.51	11.46	4.34	4.80	10.93

## Discussion

The MTHFR gene encodes for a protein with important enzymatic functions in methyl-group metabolism. Numerous studies have addressed the correlation of MTHFR polymorphisms and risk of cancers. On the other hand, the role of persistent hrHPV infections in the development of cervical precursor lesions and cancer is unequivocally demonstrated 27.

The impairment of MTHFR enzymatic activity through *C677T* polymorphism was frequently associated with different type of malignancy 28–30, but its link to cervical cancer is still under debate, the results being controversial. Whereas some current meta-analysis suggested that *C677T* MTHFR polymorphism may not be associated with cervical cancer 31, other studies found a correlation (without statistically robustness) between mutant *C677T* in white women and low risk of cervical cancer 32. Further meta-analysis highlights the association between *C677T* MTHFR polymorphism and cervical cancer, emphasizing that this association may be race specific 33. Taking into account the geographical variability in the MTHFR polymorphisms we found a prevalence of TT homozygosity (*C677T* polymorphism) in our samples of around 6.67%, which is in agreement with data published by Osian *et al*. 34. Still, for CT heterozygosity, we found a higher prevalence (43.33%) in our group. The analysis of *C677T* polymorphism in correlation with the severity of cervix lesion associate the presence of T allele with cervical cancer susceptibility (*P* = 0.0035). Our results are similar with the study of Goodman *et al*. 35, but are in contrast with those of Lambropoulos *et al*.^36^. Goodman *et al*. reported that women with CT genotype had twice the risk of cervical LGSILs, whereas TT genotype women had almost three times the risk of LGSILs compared with women with the homozygous MTHFR CC genotype 35.

We investigated patients with different cytology, including cancer. Overall, for both CT and TT genotypes we found a threefold increased risk for cervical lesion development. These observations are in contrast with Agodi *et al*. 37, who reported that the *677T* allele of MTHFR is a protective factor against cervical carcinogenesis. Moreover, the results from our investigation support the hypothesis that individuals carrying the *T* allele are susceptible for acquiring HPV cervix infection (*P* = 0.0287). The susceptibility of *C667T* mutation carriers individuals to HPV infection is due to genomic instability 21 created by decreased enzyme activity. This is an important finding which suggests that MTHFR polymorphism do not cause only cancer susceptibility but also for acquiring viral infection.

In our study, HPV-positive women who had at least one variant of *T* allele (*C677T* polymorphism) had a 14-fold increased risk (*P* < 0.001, OR-14.22) for cervical dysplasia compared with HPV-negative women with the CC genotype. On the other hand, the *T* allele is almost sixfold more frequent in patients with tumours than in control group (*P* = 0.0084, OR = 5.667). No association between HPV types (high or low risk) and MTHFR polymorphisms was found. Mei *et al*. highlighted that the association between *C677T* MTHFR polymorphism and cervical cancer could be affected by HPV status. *C677T* polymorphism might lead to cervical cancer by impairing DNA methylation in both host and viral genomes 33.

On the other hand, our findings concerning the Romanian women assert the results of Kang *et al*. 38, who did not found any association between MTHFR *A1298C* polymorphism and cervical cancer. Gathering these findings, we support the idea that MTHFR polymorphism presence could represent a risk factor for cervical HPV acquisition and persistence. Furthermore, it is assumed that MTHFR plays an important role in DNA methylation, an important epigenetic mechanism which modulates gene expression. Differences in genome-wide methylation are linked with pathological conditions, including cervical neoplasia 39. As aberrant methylation of tumour-related genes has been recognized as an important mechanism associated with the development and progression of cancer 40, methylation of CpG islands from MTHFR gene promoter is also a result of the oncogenesis process. Thus, the MTHFR methylation analysis can be included in a panel of hypermethylated genes, which may be useful for a sensitive and specific diagnosis of cervical lesions.

We found that MTHFR hypermethylation is rare in LGSIL lesion, but more frequent in HGSIL and cancer lesions. Significant differences were observed between HPV-positive samples, control group and HPV-negative dysplastic lesions (*P* < 0.0001). These findings support the involvement of papillomaviruses in oncogenesis, a process associated with aberrant methylation. No association between MTHFR genotype and MTHFR methylation was found. Therefore, we concluded that MTHFR polymorphism is a factor for promoting cervical dysplasia, but promoter methylation might result from cervical lesion severity linked to viral infection. This scenario is supported by the fact that the methylation level of MTHFR seems to vary between high-risk and low-risk HPV types. Moreover, the most powerful methylator HPV type is HPV16, in single- and co-infections. To our knowledge, these data has not been reported before.

## Conclusion

Our study shows that the *C677T* and not the *A1298C* MTHFR polymorphism represents a risk factor of cervical dysplasia. MTHFR promoter methylation varies between high-risk and low-risk HPV types, with HPV 16 being linked to most methylation changes in the promoter. No link between MTHFR polymorphism at *C677* and MTHFR promoter methylation was observed. Given that in Romania a high prevalence of HPV infection is found, our patient cohort might prove valuable for studies of the dynamics of MTHFR promoter methylation.
